# Self-Organizing Maps of Molecular Descriptors for Sesquiterpene Lactones and Their Application to the Chemotaxonomy of the Asteraceae Family

**DOI:** 10.3390/molecules17044684

**Published:** 2012-04-20

**Authors:** Marcus T. Scotti, Vicente Emerenciano, Marcelo J. P. Ferreira, Luciana Scotti, Ricardo Stefani, Marcelo S. da Silva, Francisco Jaime B. Mendonça Junior

**Affiliations:** 1 Universidade Federal da Paraíba, Departamento de Engenharia e Meio Ambiente, Campus IV, 58297-000, Rio Tinto, PB, Brazil; 2 Institute of Chemistry, University of São Paulo, Postal Box 26777, São Paulo, 05513-970, Brazil; 3 Universidade Presbiteriana Mackenzie, Cep 01302-907, São Paulo, Brazil; 4 Universidade Federal da Paraíba, Centro de Biotecnologia, Campus I 50670-910, João Pessoa, PB, Brazil; 5 Universidade Federal de Mato Grosso, Instituto de Ciências Exatas da Terra – ICET, Rodovia MT-100 Km 3,5, 78698-000, Pontal do Araguaia, MT, Brazil; 6 Universidade Estadual da Paraíba, Departamento de Ciências Biológicas, Laboratório de Síntese e Vetorização de Moléculas, 58070-450, João Pessoa, PB, Brazil

**Keywords:** astearaceae, chemotaxonomy, sesquiterpene lactones

## Abstract

The Asteraceae, one of the largest families among angiosperms, is chemically characterised by the production of sesquiterpene lactones (SLs). A total of 1,111 SLs, which were extracted from 658 species, 161 genera, 63 subtribes and 15 tribes of Asteraceae, were represented and registered in two dimensions in the SISTEMATX, an in-house software system, and were associated with their botanical sources. The respective 11 block of descriptors: Constitutional, Functional groups, BCUT, Atom-centred, 2D autocorrelations, Topological, Geometrical, RDF, 3D-MoRSE, GETAWAY and WHIM were used as input data to separate the botanical occurrences through self-organising maps. Maps that were generated with each descriptor divided the Asteraceae tribes, with total index values between 66.7% and 83.6%. The analysis of the results shows evident similarities among the Heliantheae, Helenieae and Eupatorieae tribes as well as between the Anthemideae and Inuleae tribes. Those observations are in agreement with systematic classifications that were proposed by Bremer, which use mainly morphological and molecular data, therefore chemical markers partially corroborate with these classifications. The results demonstrate that the atom-centred and RDF descriptors can be used as a tool for taxonomic classification in low hierarchical levels, such as tribes. Descriptors obtained through fragments or by the two-dimensional representation of the SL structures were sufficient to obtain significant results, and better results were not achieved by using descriptors derived from three-dimensional representations of SLs. Such models based on physico-chemical properties can project new design SLs, similar structures from literature or even unreported structures in two-dimensional chemical space. Therefore, the generated SOMs can predict the most probable tribe where a biologically active molecule can be found according Bremer classification.

## 1. Introduction

The Asteraceae family is one of the largest families of angiosperms in the World. The main secondary metabolites isolated from the species of Asteraceae are monoterpenes, sesquiterpenes, sesquiterpene lactones—SLs [1], polyacetylenes [2], flavonoids [3,4], benzofurans and benzopyrans [5], coumarins [6], diterpenoids [7] and triterpenoids [8]. All of the classes currently have a number of representatives (at least several hundred) that are considered to be satisfactory for studying the chemotaxonomy. 

Approximately 23,000 species of this family have been botanically described, and several reviews have been published on their chemistry and biology [9,10,11]. This family has been classified by various botanists [12,13,14,15,16,17,18,19].

The first classifier was Cassini, who published a diagram in 1816 showing the interrelationships of 19 tribes [12]. In 1976, Carlquist [15] divided the Asteraceae into two subfamilies, based on morphological studies: Asteroideae and Cichorioideae. Wagenitz [16] also proposed a division, in the same year, into two subfamilies that differed from Carlquist by including the Eupatorieae tribe in the Asteroideae instead of the Cichorioideae. This diphyletic view of the family was a major step in understanding the relationships between the Asteraceae tribes. 

**Figure 1:**
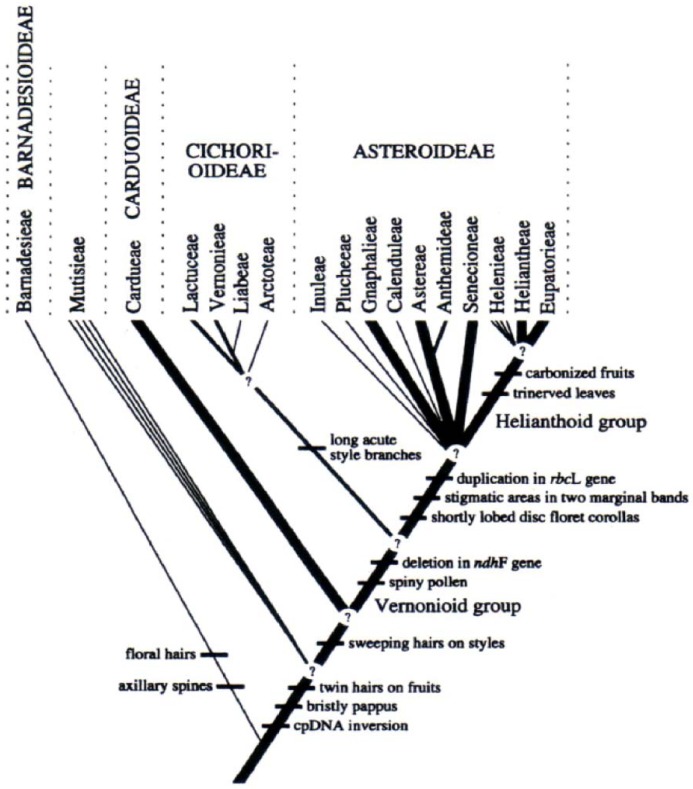
Phylogenetic diagram of the Asteraceae tribes, according to Bremer [18].

In 1987, Bremer presented a cladogram of the Asteraceae that was based on 81 characters, 10 of which were chemical [17]. The remaining characters were mostly morphological and DNA characteristics. This study is an example of classification that incorporates chemical characters combined with morphological and molecular characters. Years later, Bremer unveiled a new classification of the Asteraceae that was based mainly on morphology, proposing four subfamilies: Asteroideae (Ast), Cichorioideae (Cic), Carduoideae (Car) and Barnadesioideae (Bar) [18]. Bremer placed the Mutiseae tribe on a branch (clade) that was not well-positioned between Barnadesioideae and Carduoideae (Figure 1).

In 2005, Funk *et al.* produced a “supertree” that showed the phylogeny of the Asteraceae family [19], using the most cited studies as well as unpublished data that were provided by contributing authors.

Secondary metabolites have a restricted distribution and specific botanical sources. In the Chemistry of Natural Products, secondary metabolites are important chemotaxonomic markers [20,21]. Chemosystematics consists of classifying organisms through chemical characteristics, thus providing some answers and/or proposals for a greater understanding of evolution [22,23,24,25]. Some chemosystematics studies tried to relate the oxidation of secondary metabolites with the phytochemical evolution and diversity based on that oxidative pathways in plants occur parallel to protective mechanisms against oxidative degradation [23,25].

There are many different types of molecular descriptors, which are often distinguished by their physico-chemical meanings or by the specific mathematical tools that are used for their calculation, such as the DRAGON v. 6.0 program [26]. The use of robust methodology to attempt to detect groupings and patterns that may be unclear even for trained human experts generally employs artificial neural networks (ANNs). ANNs are defined as computational models with structures that are derived from a simplified concept of the brain, in which a number of nodes, called neurons, are interconnected in a network-like structure. Because ANNs are not restricted to linear correlations and are capable of considering non-linear data correlations, they can be used efficiently for modelling, prediction and classification. The most commonly used ANN architecture for pattern recognition and classification is the self-organising map (SOM). This method can map multivariate data onto a two dimensional grid, grouping similar patterns near to each other [27,28]. In natural product chemistry there are few works applications of ANNs for classification of Asteraceae tribes using SLS [29,30]. Da Costa and coworkers used SOM with Radial Distribuction Function (RDF) descriptors of a set of 144 SLs of three tribes (Eupatoriae, Heliantheae and Vernonieae) of the family Asteraceae [29]. Hristozov and co-workers used two different molecular descriptors: Atom counts and with two supervised methods—counterpropagation neural networks and k-nearest neighbors (k-NN) [30]. 

In the present study, 1,111 SLs, which were extracted from 658 species, 161 genera, 63 subtribes and 15 tribes of the Asteraceae family, were registered in two dimensions using SISTEMATX, our in-house databank software, which allowed a chemotaxonomic analysis among the tribes of Asteraceae family, employing molecular descriptors (including average degree of oxidation), multiple linear regression and unsupervised neural networks. The results are compared with the systematic classifications proposed by Bremer [18]. Therefore, this work has the following objectives: (1) To compare the classification using SLs as chemical markers with the morphological and molecular classification used in the Bremer tree. Such models based on physico-chemical properties can project new design SLs, similar structures from literature or even unreported structures in two-dimensional chemical space. Therefore, the generated SOMs can predict the most probable tribe where a biologically active molecule can be found according Bremer classification. (2) To verify the relationship between the degree of oxidation of the sesquiterpene lactones and the evolution of the Asteraceae tribes.

## 2. Results and Discussion

### 2.1. Data Generated from the Three-Dimensional Structures and Their Respective Chemical Occurrences, Using SISTEMATX

Data shown in Table 1 were obtained after the generation of botanical data. There are 1111 different sesquiterpene lactones, which correspond to 1979 chemical occurrences for 15 tribes, 63 subtribes, 161 genera and 658 species of the Asteraceae family.

**Table 1:** Tribes listed following tribe’s subfamily according Bremer [18], respective acronyms, botanical data and chemical occurrences added and used in the SISTEMATX.

Tribe	Acronym	Subtribe	Genus	Species	Occurrences	Compounds	Occurrences/compounds
Mutisieae	Mut	2	7	11	27	22	1.23
Cardueae	Car	3	15	55	118	63	1.87
Lactuceae	Lac	5	6	13	28	17	1.64
Vernonieae	Ver	7	16	85	215	116	1.85
Liabeae	Lia	1	4	7	15	12	1.25
Arctoteae	Arc	3	4	7	14	12	1.16
Inuleae	Inu	1	5	21	104	69	1.51
Plucheeae	Plu	1	1	1	1	1	1.00
Gnaphalieae	Gna	3	5	5	7	7	1.00
Astereae	Ast	2	2	3	8	8	1.00
Anthemideae	Ant	9	15	130	363	154	2.16
Senecioneae	Sen	2	9	22	57	37	1.54
Helenieae	Hel	3	14	73	209	123	1.69
Heliantheae	Hln	9	39	163	612	385	1.59
Eupatorieae	Eup	12	19	62	201	165	1.22
Total		63	161	658	1979	1111	1.78

We define number of occurrences for a superior taxon, counting how many times a compound appears in determined species belonging to that taxon. The Heliantheae was found to be the tribe with the greatest number of genera, species, occurrences and compounds. Other tribes with large numbers were the Anthemidae, Eupatorieae, Vernonieae and Helenieae.

### 2.2. Molecular Descriptors and Average Degree of Oxidation (NOX/nC)

The 1,111 molecules obtained were used as input data in the DRAGON 6.0 program for calculating the descriptors. During the pre-treatment of data, constant variables were excluded for each block of descriptors, as well as those that presented the same value except in one case (near constant variable). For the remaining descriptors, pairwise correlation (r < 0.99) analysis was performed to exclude those highly correlated.

After the values of the descriptors were calculated, each of the 11 generated files, one for each block of descriptor, was attached to its respective botanical occurrence (tribe, subtribe, genus) and to its degree of oxidation (NOX/nC), *i.e.*, the value equal to the number of oxidations divided by the number of carbon atoms present in each molecule. Subsequently, the mean of the descriptor values and the degree of oxidation was calculated for each tribe. The resulting file was used as input data for MobyDigs 1.1, to select the descriptors and to generate multiple linear equations. 

Several statistically significant equations were obtained for each block of descriptors, to explain the variance in the values of the degree of oxidation between tribes. The equations yielding the highest values of Q_cv_^2^ with only one descriptor were selected, as follows:

[Nox/nC = 0.501 (±0.042) AMW − 4.209 (0.290) 

(n = 15; r^2^ = 0.981; s = 0.019; F = 659.76; Q_cv_^2^ = 0.975; SPRESS = 0.020)]    (1) 

[Nox/nC = 0.300 (±0.065) L2v − 1.659 (±0.198) 

(n = 15; r^2^ = 0.883; s = 0.046; F = 98.22; Q_cv_^2^ = 0.857; SPRESS = 0.047)]     (2)

[Nox/nC = 1.789 (±0.413) H5m − 1.060 (±0.075) 

(n = 15; r^2^ = 0.871; s = 0.049; F = 87.50; Q_cv_^2^ = 0.839; SPRESS = 0.050)]     (3)

Equation (1) contains a constitutional descriptor (AMW) and has high values of statistical coefficients (r^2^, Q_cv_^2^ and F). This descriptor represents the mean molecular weight, including the atomic weights of the molecule. Sesquiterpene lactones mostly present only carbon, hydrogen and oxygen atoms; SLs with few hydrogen atoms, more double bonds and more oxygen atoms have larger AMW values, and consequently, have higher values for the degree of oxidation (NOX / nC).

**Figure 2:**
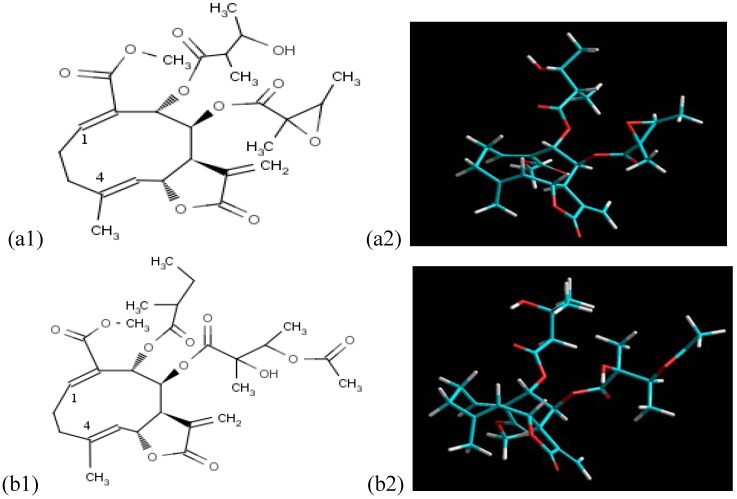
Structures in 2D (two dimensions) of 2 melampolides teraludin I (a1) and tetrahelin C (b1), and respective 3D (three dimensions) structures (a2 and b2).

A WHIM (Weighted Holistic Invariant Molecular) (L2v) descriptor is present in equation (2), which also showed high statistical coefficients. The L2v descriptor is the second eigenvalue of the covariance matrix of the molecule atomic coordinates, weighted by the van der Waals volume, and it accounts for the molecular size along the second principal direction. The SLs with an inverted configuration of the double bonds of the ring, in other words, Z and E configurations on the positions 1 and 4 respectively, show branches with angles that are closer to 90° relative to the plane of the ring. These ramifications were found to be rich in esters and hydroxyls (Figure 2). Moreover, epoxidised SLs were found to have high L2v values. 

Equation (3) contains a GETAWAY (Geometry, Topology, and Atom-Weights Assembly) descriptor (H5m). The degree of accessibility between the atoms that are separated by a topological distance of 5 and their respective atomic masses, are positively related to the value of H5m. Compounds with higher value of this descriptor have greater accessibility between oxygen with topological distance of 5. Considering a graph representation of a compound, topological distance is the number of edges (bonds) between two vertices (atoms), Sesquiterpene lactones with ester-rich ramifications have high H5m values. Table 2 was obtained through equation (1), which yields the best statistical indices. 

It is evident, observing the degree of oxidation for 15 tribes (Table 2), that the Cichorioideae and Asteroideae tribes did not cluster and apparently, present no relationship between the degree of oxidation of the sesquiterpene lactones and the evolution of the tribes and for this family, which produce several metabolites classes, evolution not only occurs through oxidation process as have reported in others studies [25].

**Table 2:** Mean values for the observed degree of oxidation (NOX/nC) for 15 tribes of the Asteraceae family and values for the degree of oxidation predicted by equation (1) and the respective residuals.

Tribe	Observed NOX/nC	Predicted NOX/nC	Residual (Actual - Calculated)
Mutisieae	−0.753	−0.774	0.021
Cardueae	−0.655	−0.626	−0.029
Lactuceae	−0.620	−0.605	−0.015
Vernonieae	−0.560	−0.582	0.022
Liabeae	−0.807	−0.772	−0.035
Arctoteae	−0.819	−0.817	−0.002
Inuleae	−0.934	−0.942	0.008
Plucheeae	−0.700	−0.723	0.023
Gnaphalieae	−0.847	−0.859	0.012
Astereae	−0.983	−0.983	0.000
Anthemideae	−0.794	−0.798	0.004
Senecioneae	−0.936	−0.929	−0.007
Helenieae	−0.698	−0.717	0.018
Heliantheae	−0.692	−0.706	0.014
Eupatorieae	−0.593	−0.603	0.009

### 2.3. Self-Organising Maps (Kohonen) and Molecular Descriptors in the Chemotaxonomy of the Asteraceae Family Tribes

The following blocks of descriptors were generated from 2D molecular structures: Constitutional descriptors, which reflecting the molecular composition of a compound, Functional groups, that are groups of atoms having a characteristic and specific reactivity, Atom-centred descriptors defined as the number of specific atom types in a molecule, BCUT (Burden Eigenvalues) are eigenvalues of a modified connectivity matrix, 2D autocorrelations are spatial autocorrelations on a molecular graph weighted by atom physico-chemical properties, Topological are descriptors based on a graph representation of the molecule [31].

Geometrical are defined in several different ways, but always derived from the 3D structure of the molecule, RDF (Radial Distribution Function) are descriptors based on a radial distribution function which can be interpreted as the probability distribution of finding an atom in a spherical volume of radius R, 3D-MoRSE (3D-Molecule Representation of Structures based on Electron diffraction) are based on the idea of obtaining information from the 3D atomic coordinates by the transform used in electron diffraction studies, GETAWAY (GEometry, Topology, and Atom-Weights AssemblY) are related to the influence of the atoms in the determination of the molecular form (leverages), and to the distance between them, WHIM (Weighted Holistic Invariant Molecular descriptors) are geometrical descriptors based on statistical indices calculated on the projections of the atoms along principal axes [31].

The 11 files of botanical occurrences were used (one for each block of descriptors) for nine tribes, along with the values of the descriptors, as input data for the SOM toolbox 2.0 software. The nine tribes selected for analysis were those with the highest numbers of botanical occurrences (Table 3). SOM projects objects (chemical occurrence) from a multidimensional space (variables—molecular descriptors) into a space of lower-dimensionality (2D plane). In this projection, the similarity relationship between objects is conserved. Thus, Kohonen networks (SOMs) can be used for clustering of objects. SOMs accomplish two things, they reduce dimensions and display similarities, which make the SOM a powerful visualization tool. The training of these networks (Kohonen) is unsupervised, that is, the investigated property is not used during the training process. Each neuron in the grid is associated with a weight, and similar patterns stimulate neurons with similar weight, so that similar patterns are mapped near each other [28].

All of the blocks of descriptors, with the exception of the constitutional (66.7%) and the 3D- MoRSe (Molecule Representation of Structure based on Electron diffraction) (67.5%) descriptors, had overall hit rates that were above 80%. The maps measured 40 by 30, with 1200 neurons, except for the maps that were obtained using functional group descriptors (35 by 35, with 1225 neurons) and GETAWAY descriptors (40 by 35, with 1400 neurons). 

In the represented maps, the chemical occurrences of certain tribes occupy regions that are labelled by the following colours:

1. Anthemideae: blue,
2. Cardueae: brown,
3. Eupatorieae: yellow,
4. Helenieae: orange,
5. Heliantheae: red,
6. Inuleae: pink,
7. Lactuceae: grey,
8. Senecioneae: light blue,
9. Vernonieae: green.

**Figure 3:**
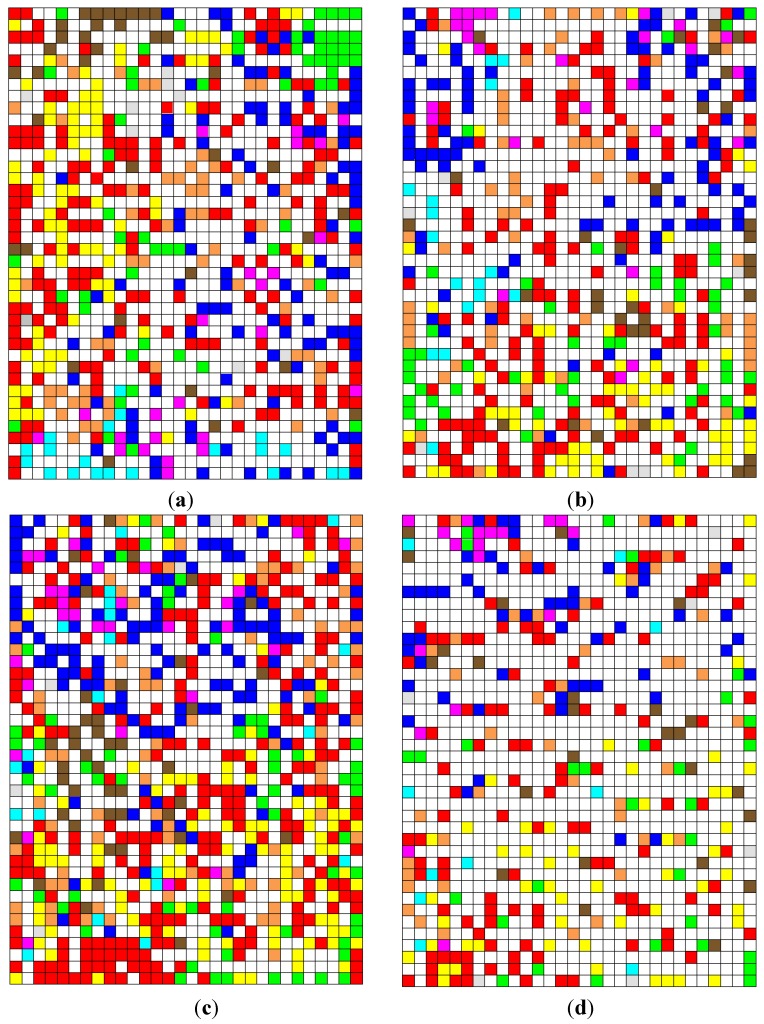
Self-organising maps obtained by classifying nine tribes of the Asteraceae family. Maps: (**a**) Using the block of atom-centred descriptors; (**b**) Using the block of BCUT descriptors; (**c**) Using the block of RDF (Radial Function Distribution) descriptors; (**d**) Using the block of 3D MoRSE descriptors.

The self-organising map (SOM) using the RDF (Radial Function Distribution) descriptors showed the largest overall hit rate (83.6%), this match rate is similar obtained by Hristozov and co-workers using the same kind of descriptors and supervised methods [30]. The SOM obtained using the constitutional descriptors had the worst hit rate. There was no significant difference regarding the percentage of hits among the other blocks of descriptors, indicating that functional groups or atom-centred descriptors, with respect to their simplicity of interpretation and acquisition, are sufficient to encode information of SLs that are differentiated according to their occurrence among the tribes of the Asteraceae family. Figure 3 shows some the most representative maps.

The Inuleae tribe had the worst hit rate; the maps could not differentiate this tribe from the others. The hit rates ranged from 27.6%, for the map that was obtained from using constitutional descriptors, to 56.7%, for the map obtained using the BCUT descriptors. This tribe’s areas (pink neurons) were close to the Anthemideae tribe (blue neurons) in all of the maps. The low hit rate of the Inuleae tribe was a result of the neurons that were occupied by this tribe, which were mixed with neurons of the Anthemideae (blue regions) and Heliantheae (regions in red) tribes (Figure 3).

**Table 3:** Results of the self-organising maps and their respective dimensions, with the values of the occurrences and the number of absolute and relative hits for the nine tribes of the Asteraceae family, using the blocks of descriptors that were generated by the DRAGON 6.0 program.

			Constitutionals – 40 × 30 ^a^		Functional groups - 35 × 35^ a^		Atom-Centred - 40 × 30^ a^		2D Autocorrelation-40 × 30^ a^	
Tribe	Occurrences		Nº of hits	% of hits	Nº of hits	% of hits	Nº of hits	% of hits	Nº of hits	% of hits
Cardueae	118		81	68.6	103	87.3	101	85.6	95	80.5
Lactuceae	28		18	64.3	20	71.4	21	75.0	14	50.0
Vernonieae	215		147	68.4	174	80.9	175	81.4	178	82.8
Inuleae	104		29	27.9	32	30.8	46	44.2	61	58.7
Anthemideae	363		268	73.8	334	92.0	338	93.1	336	92.6
Senecioeae	57		38	66.7	50	87.7	53	93.0	47	82.5
Helenieae	209		110	52.6	159	76.1	171	81.8	176	84.2
Heliantheae	612		451	73.7	517	84.5	511	83.5	522	85.3
Eupatorieae	201		130	64.7	148	73.6	157	78.1	151	75.1
Total	1907		1272	66.7	1537	80.6	1573	82.5	1580	82.9
			**BCUT - 40 × 30^ a^**		**Topological - 40 × 30^ a^**		**Geometrical - 40 × 30^ a^**		**RDF - 40 × 30^ a^**	
**Tribe**	**Occurrences**		**Nº of hits**	**% of hits**	**Nº of hits**	**% of hits**	**Nº of hits**	**% of hits**	**Nº of hits**	**% of hits**
Cardueae	118		105	89.0	99	83.9	96	81.4	97	82.2
Lactuceae	28		19	67.9	22	78.6	20	71.4	17	60.7
Vernonieae	215		165	76.7	168	78.1	164	76.3	167	77.7
Inuleae	104		59	56.7	57	54.8	49	47.1	54	51.9
Anthemideae	363		342	94.2	324	89.3	339	93.4	341	93.9
Senecioeae	57		52	91.2	46	80.7	44	77.2	48	84.2
Helenieae	209		183	87.6	175	83.7	180	86.1	180	86.1
Heliantheae	612		513	83.8	497	81.2	487	79.6	529	86.4
Eupatorieae	201		154	76.6	160	79.6	156	77.6	161	80.1
Total	1907		1592	83.5	1548	81.2	1535	80.5	1594	83.6
			**3D-MoRSE-40 × 30 ^a^**		**GETAWAY- 40 × 35 ^a^**		**WHIM – 40 × 30 ^a^**				
**Tribe**	**Occurrences**		**Nº of hits**	**% of hits**	**Nº of hits**	**% of hits**	**Nº of hits**	**% of hits**		
Cardueae	118		83	70.3	104	88.1	106	89.8		
Lactuceae	28		17	60.7	17	60.7	19	67.9		
Vernonieae	215		143	66.5	164	76.3	166	77.2		
Inuleae	104		43	41.3	50	48.1	57	54.8		
Anthemideae	363		290	79.9	341	93.9	339	93.4		
Senecioeae	57		33	57.9	46	80.7	44	77.2		
Helenieae	209		121	57.9	173	82.8	158	75.6		
Heliantheae	612		430	70.3	498	81.4	517	84.5		
Eupatorieae	201		128	63.7	168	83.6	149	74.1		
Total	1907		1288	67.5	1561	81.9	1555	81.5		

**^a^** blocks of descriptors used and dimensions of the self-organising maps.

The Anthemideae tribe had the highest hit rates, the lowest value of 73.8% on the map using constitutional descriptors and the highest value obtained with the BCUT descriptors (94.2%). In all of the maps that were obtained, the regions occupied by this tribe (in blue) were different from those for the Eupatorieae (yellow), Vernonieae (green) and, to a lesser extent, the Heliantheae (red) and Helenieae (orange) tribes (Figure 3).

The Senecioneae tribe had high hit rates, which were above 75%, except for the maps that were obtained by using the constitutional and 3D-MoRSE descriptors. In most of the maps, the regions occupied by this tribe (light blue) were near to the Anthemideae (blue) tribe, which agrees with the classifications proposed by Bremer, Jansen and Funk.

The Heliantheae tribe had high values of hit rates in all of the maps. This tribe had the highest number of occurrences and compounds in the present study, and its structural diversity, in terms of SLs, is displayed in the self-organising maps. Despite the fact that it is mainly concentrated in a specific region (red), this tribe occupies an extensive area in the SOMs. As previously mentioned, the regions of the Heliantheae tribe are close to those of the Helenieae (orange) and Eupatorieae (yellow) tribes (Figure 3).

The Vernonieae tribe generated high hit rates, except for the maps that use constitutional (68.4) and 3D-MoRSE (66.5) descriptors. With the exception of the map that used the atom-centred block of descriptors, no proximity was noted between the Vernonieae tribe and the Lactuceae (grey), which also belongs to the same subfamily (Cichorioideae). This observation can be explained by the low number of occurrences (28) and compounds (17) of the Lactuceae tribe used in this study. This result helps elucidate the low hit rates that this tribe achieved in all of the maps, except for in the maps that used functional groups (71.4%) and topological (78.6%) and geometrical (71.4%) descriptors. This tribe (grey) occupied few neurons that are distributed in all of the maps, thus preventing the determination of a predominant region (Figure 3).

The Cardueae tribe (subfamily Carduoideae) presented high hit rates in all of the maps, except in the SOM obtained from using constitutional descriptors (68.6%). The regions that are occupied by this tribe (brown) were distributed between the Heliantheae (red), Eupatorieae (yellow), Vernonieae (green) and Anthemideae (blue) tribes.

The prediction performance of the most significant block of descriptors 2D and 3D, atom-centred and RDF, respectively for the nine training set and test set generated from original set, are shown on Table 4 and Table 5. 

**Table 4:** Summary of training and test match results (%) using atom-centred descriptors.

Tribe	*Train set 1*	*Train set 2*	*Train set 3*	*Train set 4*	*Train set 5*	*Train set 6*	*Train set 7*	*Train set 8*	*Train set 9*	*Average*
Cardueae	89.1	79.4	86.9	86.1	84.6	88.9	85.2	90.7	91.7	**87.0**
Lactuceae	68.0	74.1	82.6	65.2	76.2	69.2	73.1	70.8	69.6	**72.1**
Vernonieae	79.1	82.4	78.9	75.1	78.3	78.6	82.4	80.0	80.2	**79.4**
Inuleae	47.8	52.7	47.3	51.6	50.0	40.9	54.8	37.5	62.5	**49.5**
Anthemideae	88.9	92.4	91.8	93.4	92.7	93.1	90.5	90.8	91.7	**91.7**
Senecioeae	87.0	82.4	82.1	85.4	87.0	88.2	82.4	86.0	84.8	**85.0**
Helenieae	75.3	74.6	72.8	76.7	70.1	71.4	78.8	77.0	75.4	**74.7**
Heliantheae	83.7	82.6	81.8	81.4	83.4	83.5	82.2	82.5	82.1	**82.6**
Eupatorieae	79.2	82.5	81.6	77.0	80.6	77.0	79.2	76.0	77.7	**79.0**
Total	81.0	80.7	80.9	80.6	81.1	80.6	81.7	80.5	81.9	**81.0**
***Tribe***	***Test set 1***	***Test set 2***	***Test set 3***	***Test set 4***	***Test set 5***	***Test set 6***	***Test set 7***	***Test set 8***	***Test set 9***	***Average***
Cardueae	62.5	68.8	81.8	76.4	42.9	30.0	80.0	63.6	100.0	**67.3**
Lactuceae	50.0	-	50.0	50.0	66.7	100.0	100.0	100.0	50.0	**70.8**
Vernonieae	63.2	59.1	68.0	66.7	52.9	85.7	72.7	75.0	85.7	**69.9**
Inuleae	9.1	30.0	33.3	20.0	27.3	20.0	20.0	14.3	42.9	**24.1**
Anthemideae	79.2	90.9	87.9	80.0	80.0	65.9	73.0	80.0	83.3	**80.0**
Senecioeae	33.3	66.7	0	66.7	66.7	83.3	83.3	71.4	80.0	**61.3**
Helenieae	57.9	75.0	62.1	45.0	44.0	54.2	67.9	59.1	91.7	**61.9**
Heliantheae	64.5	66.7	58.5	60.9	70.4	70.7	65.0	62.3	83.9	**67.0**
Eupatorieae	72.2	27.8	54.5	55.6	80.0	56.5	39.3	55.6	66.7	**56.5**
Total	64.2	64.2	64.2	61.6	63.7	62.6	63.7	64.2	82.1	**65.6**

**Table 5:** Summary of training and test match results (%) using RDF descriptors.

Tribe	*Train set 1*	*Train set 2*	*Train set 3*	*Train set 4*	*Train set 5*	*Train set 6*	*Train set 7*	*Train set 8*	*Train set 9*	*Average*
Cardueae	82.7	82.4	87.2	87.3	82.5	88.2	86.5	79.8	83.7	**84.5**
Lactuceae	50.0	33.3	46.2	42.3	42.3	44.0	50.0	41.7	45.8	**44.0**
Vernonieae	78.8	76.8	75.9	73.5	74.9	78.8	78.6	78.5	77.8	**77.1**
Inuleae	62.2	52.8	53.3	59.8	61.5	67.4	45.2	56.8	50.0	**56.6**
Anthemideae	93.3	95.7	93.8	93.7	93.4	89.2	89.5	91.0	92.5	**92.5**
Senecioeae	71.2	77.4	77.4	81.1	82.4	89.6	75.5	83.3	76.6	**79.4**
Helenieae	76.5	71.4	79.9	72.8	75.0	77.7	80.1	75.0	72.9	**75.7**
Heliantheae	84.9	81.3	82.9	82.0	81.2	84.9	81.9	83.8	84.7	**83.1**
Eupatorieae	77.6	77.7	73.3	78.1	82.4	66.8	72.5	74.9	73.9	**75.2**
Total	81.7	79.9	80.7	80.3	80.6	81.1	79.5	80.3	80.4	**80.5**
**Tribe**	***Test set 1***	***Test set 2***	***Test set 3***	***Test set 4***	***Test set 5***	***Test set 6***	***Test set 7***	***Test set 8***	***Test set 9***	***Average***
Cardueae	87.5	70.0	77.8	62.5	73.3	50.0	42.9	71.4	57.1	**65.8**
Lactuceae	0	50.0	50.0	50.0	0	66.7	50.0	25.0	25.0	**35.2**
Vernonieae	63.6	58.8	56.3	42.3	80.0	61.5	56.5	50.0	52.4	**57.9**
Inuleae	33.3	40.0	33.3	28.6	62.5	44.4	45.5	44.4	37.5	**41.1**
Anthemideae	68.8	77.8	81.4	84.4	87.2	65.5	76.7	66.7	73.3	**75.8**
Senecioeae	80.0	25.0	50.0	50.0	66.7	77.8	75.0	66.7	40.0	**59.0**
Helenieae	54.8	54.2	65.0	44.4	61.9	47.6	52.2	44.0	64.3	**54.3**
Heliantheae	59.1	63.6	71.0	61.6	50.0	60.3	61.5	48.0	71.4	**60.7**
Eupatorieae	44.4	35.3	60.0	70.0	35.7	29.4	63.2	36.4	23.8	**44.2**
Total	59.6	59.6	67.4	60.6	64.8	56.5	60.1	51.8	56.0	**59.6**

Table 4 presents significant match on training and test set. The performance of the test sets (65.6%) is almost 15% lower than the training (81.0%), but significant (65.6%). Inulieae and Eupatoriae have lower match values on training models on Table 3 and show the same performance on test results. Inuleae is the only one that the prediction is insignificant (lower than 50%). The test results for the atom-centred descriptors are slight higher than SOMs using RDF (Table 5) which have the overall.

The chemical pattern of SLs from Asteraceae family, obtained by using molecular descriptors and neural networks is a powerful tool for bioprospecting studies. Since these models are built based on physico-chemical properties, some SLs proposed and design based on QSAR models [32,33,34,35,36,37,38,39] of drug design studies can be projected in these SOMs, which provide the following information: association of similar compounds with potential analogous biological activities and the probable tribe that these structures can be isolated. Some models with the same or similar tasks were proposed in literature as already cited work Hristozov and coworkers [30] and the “ChemGPS Chemical Space” proposed by Larsson and coworkers [40]. 

## 3. Experimental

### 3.1. Acquisition and Registering of Structures of Sesquiterpene Lactones and Their Respective Botanical Occurrences

Data on the structure of the investigated molecule and its respective botanical occurrences were added to the SISTEMATX, based on a literature review [1]. In the SISTEMATX, the molecule must be associated with its class (sesquiterpene lactones) and with the respective skeleton. The botanical record is made in the “Botanical Data” module of the SISTEMATX. For the present study, Bremer’s classification was followed [18]. In the SISTEMATX program, a new version of SISTEMAT [41], compounds are drawn in two dimensions. For the chemotaxonomic studies nine tribes with higher values of botanical occurrence were used (>50). Lactuceae tribe was included in the studies for two reasons: to analyse the chemical pattern with Vernonieae tribe (both belong to Cichorioideae subfamily) and, have a significant value of botanical occurrence/compounds (>1.5). 

### 3.2. Acquisition of Three-Dimensional Structures of Sesquiterpene Lactones and Exportation of Botanical Data

The 3D coordinates of the SLs were generated by the SISTEMATX program, based on the 2D constitution data of the molecules that were drawn directly into the system, with the “Export Botanical Data” module, using the CORINA 3.2 software [42]. Three-dimensional structures generated can be saved in files under the formats .mol or .hin, thus allowing them to be used as input data for the generation of various molecular descriptors. All of the registered sesquiterpene lactones from the Asteraceae family were selected. The molecules were saved in MDL format files (.mol).

### 3.3. Molecular Modelling

Molecular modelling computations were performed on Hyperchem 8.0 for Windows [43]. The molecules were subjected to geometry optimisation and conformational analysis. The rotatable cyclic bonds were included as variable torsions and allowed to be changed simultaneously in the conformational search using Hyperchem’s force field MM+. The search was performed applying a usage-directed search method which was terminated after energy minimization of 500 unique starting geometries. The resulting structures were energy minimized using semi-empirical quantum chemical method Austin model 1 (AM1), and the value of 0.001 kcal/mol was used for the root mean square gradient termination condition [44,45]. 

The molecules were saved as .mol (mdl) files, to compute various molecular descriptors using DRAGON Professional v. 6.0 [26].

### 3.4. Molecular Descriptors

Descriptors provided by DRAGON were calculated, with a total of 1281 descriptors, including the following 11 block of descriptors: Constitutional, Functional groups, BCUT, Atom-centred, 2D autocorrelations Topological, Geometrical descriptors, RDF (Radial Function Distribution) descriptors, 3D-MoRSE descriptors (Molecule Representation of Structure based on Electron diffraction), GETAWAY (Geometry, Topology, and Atom-Weights Assembly) and WHIM (Weighted Holistic Invariant Molecular) [31,46,47,48,49,50,51].

Constant variables were excluded for each block of descriptors as well as those that presented only one different value in the series. For the remaining descriptors, pairwise correlation (r < 0.99) analysis was performed to exclude those that were highly correlated. The remaining descriptors, 32 from Constitutional, 35 from Functional groups, 40 from BCUT, 42 from Atom-centred, 38 from 2D autocorrelations, 59 from Topological, 42 from Geometrical, 150 from RDF, 160 from 3D-MoRSE, 188 from GETAWAY and, 99 from WHIM, were submitted to statistical analysis.

### 3.5. Calculation of Multiple Linear Regression

The MobyDigs 1.1 program [52] was used for the calculation of MLR models using a Genetic Algorithm (GA) [53]. Given the need to select the most statistically significant models, the search for the best models is usually performed using the ordinary least squares regression under the GA approach, *i.e.*, through the genetic algorithm variable subset selection method. In the GA terminology, a population is characterised by a set of candidate variables (the genetic heritage of the population) and is composed of individuals, meaning that models are made of one or more populations of variables. The parameters used in GA setup of the MobyDigs program were: maximum number of models in a population = 50; maximum number of variables in a model = 3; trade-off parameter = 0.5 (both crossover and mutation are taken into account and the role played by the two processes is equally balanced); number of the best models for each size = 3; selection pressure (bias) = 0.5. 

### 3.6. Self-Organising Maps

The whole dataset was presented to the network before any adjustments were made. In each training step, the dataset was partitioned according to the Voronoi regions of the map weight vectors. The neural network was trained until its convergence to minimal error. The correct prediction of these groups and the total correct prediction of the compounds were subsequently evaluated. All of the DRAGON descriptors that were previously selected by pairwise correlation analysis were analysed with SOMs in Matlab 6.5 and the SOM Toolbox 2.0 [54,55,56].

The SOM toolbox is a set of Matlab functions that can be used for the creation, visualisation and analysis of self-organising maps. The literature shows that the determination of the size of an SOM is an empiric process. Initially, a heuristic formula of m = 5.(n)^0.5^ is typically used for the total number of map units, where n is the number of samples [28]. For the most significant models, to evaluate the prediction ability, the set was split into training and test set. The size of the test set was around 10% of the whole set, randomly selected, and it was generated nine series. This is important because there must be a trend between the learning of the network and its ability of prediction that is verified in the results. Training and test performance are evaluated by computing the ratio of the number of samples correctly classified by SOM. 

## 4. Conclusions

No relationship could be established between the degree of oxidation of the sesquiterpene lactones and the evolution of the Asteraceae tribes. Several equations had highly significant coefficients, with only one descriptor of several blocks. Some structural features, which were related to the degree of oxidation, could be identified by interpreting molecular descriptors.

The self-organising maps obtained for the nine tribes, except Inuleae, had high hit rates and separated the tribes according to previously proposed classifications when using molecular descriptors. SOMs generated from atom-centred and RDF descriptors yield most significant results and can be used as a classification tool for lower hierarchical levels, such as tribes. Since these SOMs are built based on physico-chemical properties, they can be used in the search for SLs with potential biological activities with the respective taxonomic information. For this propose the authors are working to increase the database and improve the models.
